# Supplementary material for: Simulating a potential mpox outbreak: Implications for control in non-endemic settings
Source: PLOS Glob Public Health. 2026 Jun 29;6(6):e0006630. doi: 10.1371/journal.pgph.0006630 (PMC13313346; doi:10.1371/journal.pgph.0006630)
Supplement: S1 Appendix — We repeat the simulations from the main paper for a population with an extended MSM subnetwork generated from a truncated power-law degree distribution. We find that our qualitative results remain unchanged, but the values of the peak of the number of active infections are lower, since single-partner nodes are effectively protected if their partner remains uninfected or recovers before transmitting the disease. We also confirm that the effects of vaccination strategies and weak workplace transmission mirror those observed in the original population. (PDF) [file pgph.0006630.s001.pdf]

S1 Appendix: Simulations for extended sexual network

We consider the same population as the one described in the main paper, but connect the MSM agents using a different MSM sexual contact network. As described in the main paper, we generate connections between the MSMs with the same power-law exponent of $\alpha = 1.5$, truncated to the range $(1, 21)$. In this appendix we show the same results as in the main paper, but for this "extended" population, shown in Fig 2B of the main paper.

In Fig S1.1, we show the total number of active cases in the population, differentiating between cases in the MSM and non-MSM populations. The results are qualitatively identical to those described in the main paper, the only difference being that the number of cases is lower. This is a consequence of the fact that the number of infected MSMs is lower in this population, since the single-partner nodes are effectively protected from the disease if their partner remains uninfected or recovers before transmitting the disease.

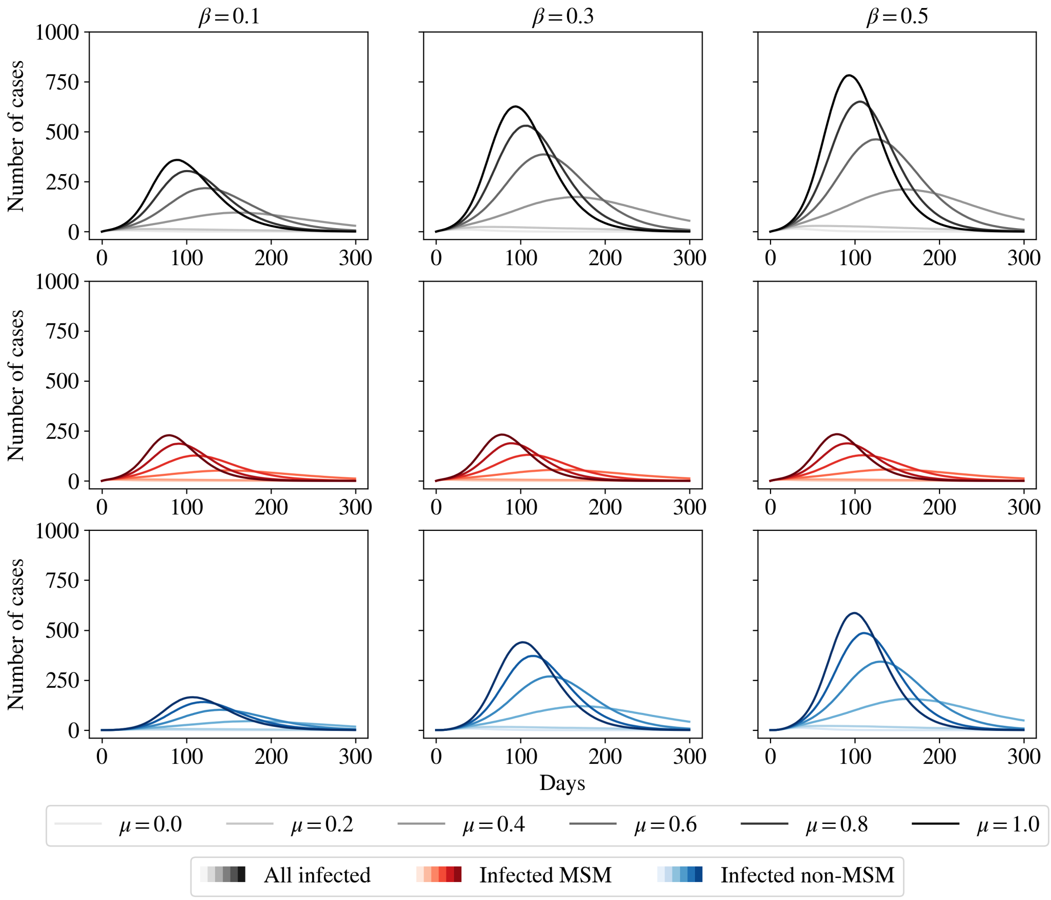

Fig S1.1: Active infections in the population. Infection curves for different values of household infectivity β and probability of sexual transmission μ, when the infection is seeded with 10 MSM agents, and the population is the extended population. Each column shows the results for different values of β, while darker colours indicate higher values of μ. The panels show the total number of active infections, and the number of active infections in the MSM and non-MSM populations respectively. These results are qualitatively identical to those quoted in the main paper, but with significantly lower peaks. The curves are averages over 500 runs.

We also consider the case of different vaccination strategies for this extended population, with the same definition of “low” and “high” risk agents, as shown in Fig 3B of the main paper. In Fig S1.2, we show the effect of ten vaccines per day on the number of active cases. As before, the effect is qualitatively identical to that described in the main paper, with the exception of the peaks of all the curves which are much lower.

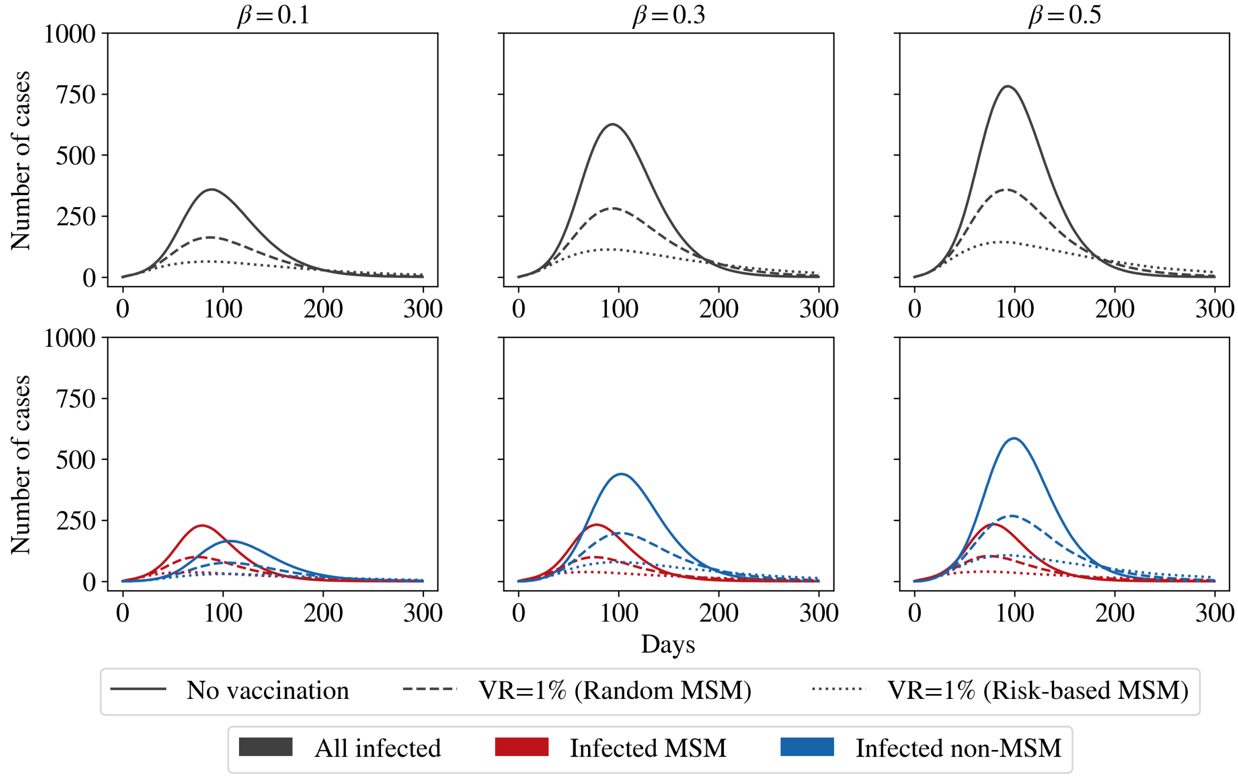

Fig S1.2: Effects of vaccination strategies on disease peak for the extended population. A vaccination rate of 10 vaccines per day (1% of MSM population) is very efficient in reducing the peak of infection, irrespective of strategy. However, the risk-based strategy of targeting “high-degree” MSMs is more efficient in bringing down the peak and shifting it earlier. These results mirror those from the original simulations. Again, the curves are averages over 500 runs.

Last, we consider the case of a small probability of workplace transmission, just as in the main paper. The results of these simulations, shown in Fig S1.3, are again qualitatively identical to those described in the main paper: if β_{work} has the relatively low value of 5% of β, a long tail of infection can be observed for higher values of β, although all peaks are lower than the corresponding values for the original population. In Fig S1.4, we show the same curves while allowing for a weak workplace transmission but setting $\mu = 0$, meaning that no spread occurs in the sexual contact network. This result is identical to Fig S7.1, as the only difference between the two populations occurs in the network structure of the MSMs.

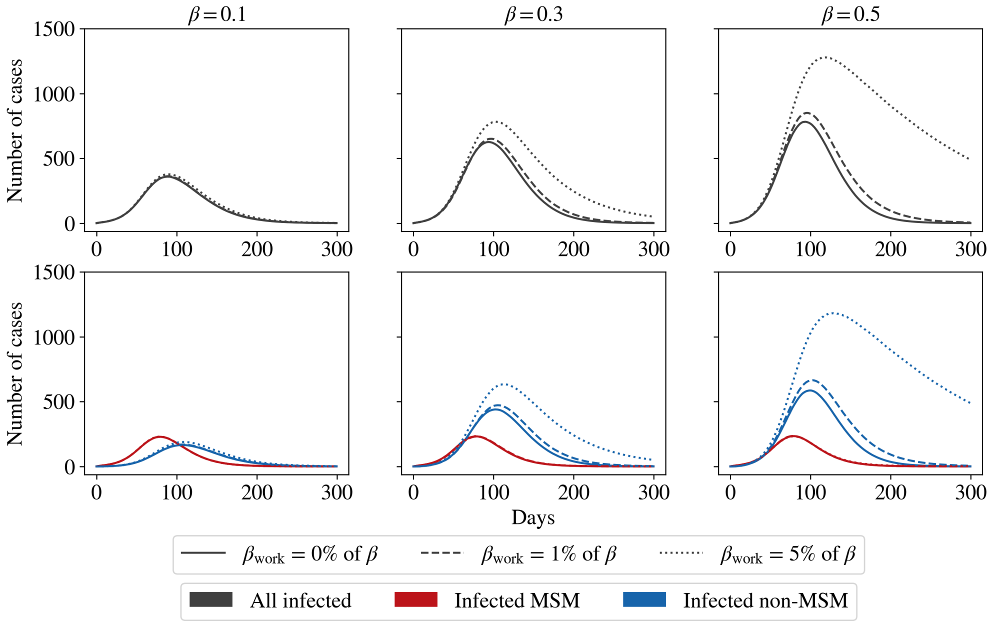

Fig S1.3: Effects of weak workplace transmission on active infections in the extended population. Just as in the original simulations, while a workplace contact multiplier of 1% does not affect the peak significantly, a multiplier of 5% leads to a much higher peak and a much heavier tail. As before, the curves are averages over 500 runs.

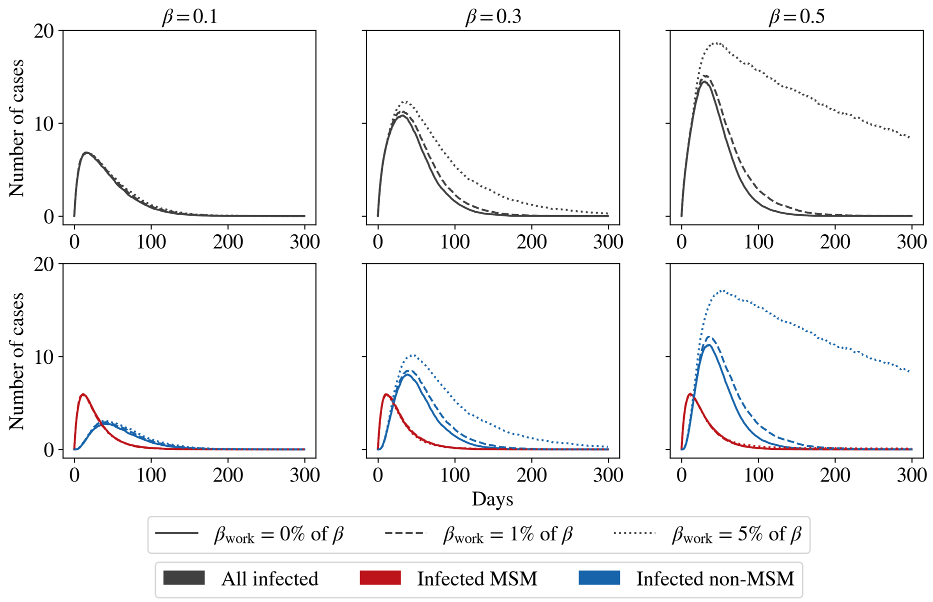

Fig S1.4: Effects of weak workplace transmission on active infections in the extended population without an underlying MSM subnetwork. We repeat the simulations of Fig S1.3, but with $\mu = 0$, meaning that the disease does not spread in the MSM subnetwork. This result is identical to that from Fig S7.1, as is expected. As before, the curves are averages over 500 runs.
