# Supplementary material for: Simulating a potential mpox outbreak: Implications for control in non-endemic settings
Source: PLOS Glob Public Health. 2026 Jun 29;6(6):e0006630. doi: 10.1371/journal.pgph.0006630 (PMC13313346; doi:10.1371/journal.pgph.0006630)
Supplement: S2 Appendix — We now study the effect of introducing female sexual partners for a subset of MSM agents in our population. Our results show that the inclusion of bisexual partnerships slows disease spread within the MSM subnetwork and consequently in the non-MSM network as well. In most parameter regimes, this also reduces the total number of non-MSM infections. However, when the household transmission parameter is low and the probability of transmission per sexual encounter is high, the increased household attack rate associated with infected female partners can instead lead to a modest rise in total non-MSM cases. (PDF) [file pgph.0006630.s002.pdf]

S2 Appendix: Effect of bisexual partnerships on disease spread

We now study the effect of introducing female sexual partners to a subset of the MSM agents in our population. Studies have shown that close to a third of the MSM population is bisexual [1,2]. Of these, around 75% have a predominant female partner who is their wife. Consequently, we have also run simulations in which 33% of MSMs are bisexual.

We begin by constructing the population (with its associated MSM subnetwork) as described in Section 2.4 of the main paper. Once this is done, we assign 33% of the agents as bisexual, indicating that in addition to their MSM contacts drawn from the distribution as described in Section 2.4, a single female household member is further designated as their female sexual partner. We assume that these female partners do not participate in additional sexual partnerships beyond their associated bisexual MSM agent and therefore do not contribute to further sexual transmission. They can, however, transmit the disease non-sexually to other household contacts.

We emphasise that this representation is intentionally simplified; we do not account for heterogeneity in the number and type of female sexual partnerships and sexual-contact frequencies. For example, Ref. [2] has shown that despite the predominant sexual partner of bisexual MSMs being their wife, 13% of MSMs have also reported sex with women outside of marriage including with commercial sex workers. We nevertheless acknowledge that incorporating such features could alter the coupling between the MSM and non-MSM networks. However, we believe this effect to be subdominant.

Sexual encounters occur as described in the main paper, however bisexual MSM agents' contacts now include their female sexual partner. Given the paucity of evidence regarding the difference in transmission between anal and vaginal routes of sexual transmission, we have assumed that the probability of sexual transmission per encounter remains the same between the MSM and their female partner as between MSMs. However, this parameter can be varied as new evidence is obtained in the future.

Our results for this population, shown in Fig S2.1, show that the introduction of bisexual partnerships in the MSM sub-network slows down the disease spread in the MSM subnetwork and consequently in the non-MSM network as well. We understand this as follows: the designation of a subset of MSMs as bisexual (with female sexual contacts) effectively reduces the probability that these MSMs can transmit the disease to their MSM partners. In our model, since the female contacts themselves do not transmit the disease sexually to anyone else, these partners effectively play the role of "dead-ends" in the MSM sexual network. Thus, using a similar argument as in Appendix S1 where we studied the extended MSM sexual network, the presence of these dead-ends slows down disease spread.

The effects of this reduction in the non-MSM network depend on the interplay of β and μ. Since the spread in the non-MSM network depends on the number of infected MSMs, this spread is correspondingly slowed down as well. In general, for almost all the parameters chosen in this work, the inclusion of bisexual MSMs leads to a corresponding reduction in the number of cases in the non-MSM network. An important exception occurs when $\beta = 0.1$ and $\mu = 1.0$. In this case, we see a modest rise in the number of cases in the non-MSM subnetwork. The explanation for this is that the inclusion of female household partners can increase the household attack rate. However, this only happens when the household transmission rate (β) is low, and the probability of sexual transmission per encounter (μ) is high. Thus, for low β and high μ, despite the slower spread, the total *number* of non-MSM cases could increase.

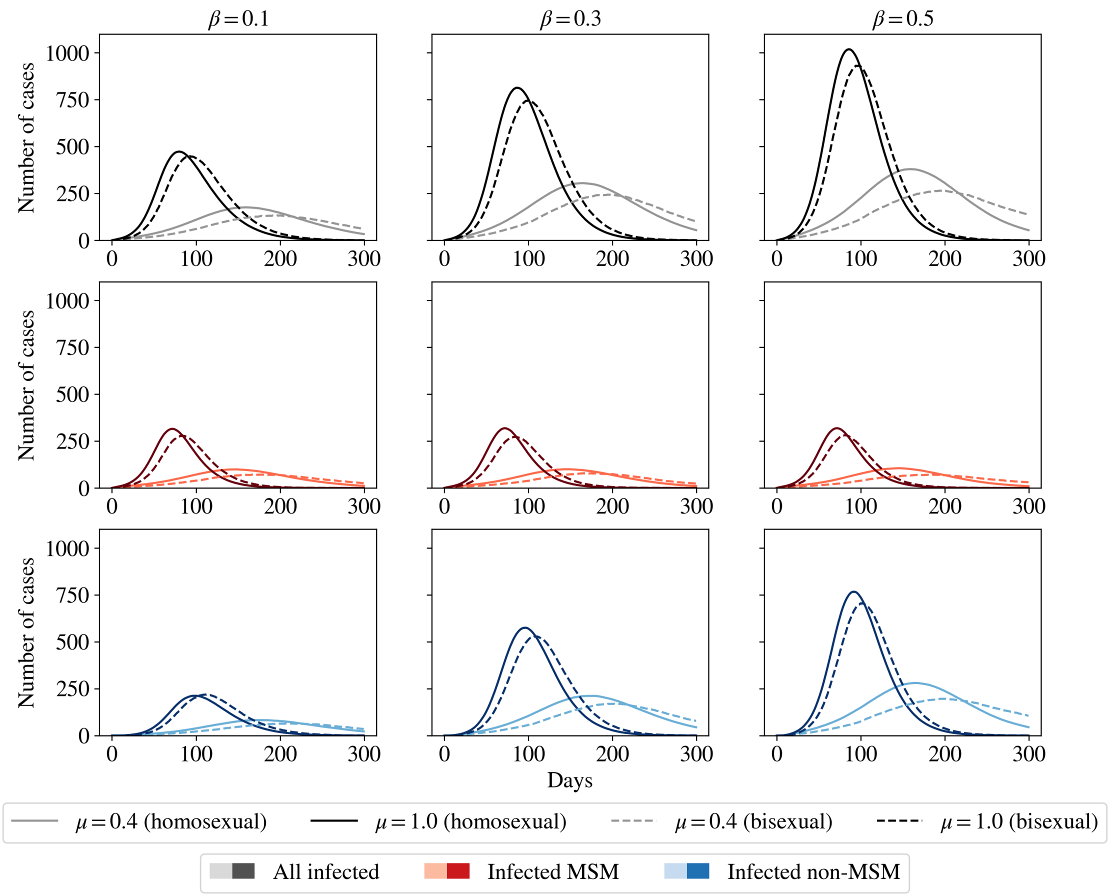

Fig S2.1: Introducing bisexual MSMs to the population. We re-run the same baseline runs from the main paper but with a population in which 33% of the MSMs have one household female sexual partner. For simplicity, we show only the results for $\mu = 0.4$ (dark lines) and $\mu = 1.0$ (faint lines) for the case in which sexual transmission amongst MSMs is exclusively homosexual (solid lines; same results as the main paper) and bisexual (dashed lines). We see that the introduction of bisexual contacts slows down the spread in the MSM-subnetwork and consequently in the non-MSM network. However, for low β and high μ this could nevertheless lead to a marginally larger number of non-MSM cases, as it effectively increases the household attack rate in MSM households.

References

[1] Ramakrishnan L, Ramanathan S, Chakrapani V, Goswami P, Deshpande S, Yadav D, et al. Comparison of Sexual Risk, HIV/STI Prevalence and Intervention Exposure Among Men Who Have Sex with Men and Women (MSMW) and Men Who Have Sex with Men Only (MSMO) in India: Implications for HIV Prevention. AIDS and Behavior. 2015;19(12):2255–2269. doi:10.1007/s10461-015-1058-2.

[2] Godbole S, Sane S, Kamble P, Raj Y, Dulhani N, Venkatesh S, et al. Predictors of Bisexual Behaviour among MSM Attending Intervention Sites May Help in Prevention Interventions for This Bridge to the Heterosexual Epidemic in India: Data from HIV Sentinel Surveillance. PLOS ONE. 2014;9(9):e107439. doi:10.1371/journal.pone.0107439.
