# Supplementary material for: Simulating a potential mpox outbreak: Implications for control in non-endemic settings
Source: PLOS Glob Public Health. 2026 Jun 29;6(6):e0006630. doi: 10.1371/journal.pgph.0006630 (PMC13313346; doi:10.1371/journal.pgph.0006630)
Supplement: S3 Appendix — We consider the scenario in which the initial infection seed occurs in the non-MSM population, and find that in order to obtain a comparable disease peak, a substantially higher number of initial infections is required. These simulations exhibit a two-peak structure, with an initial non-MSM-driven outbreak followed by a second peak once transmission enters the MSM subnetwork. (PDF) [file pgph.0006630.s003.pdf]

S3 Appendix: Simulations for an initial infection seed of non-MSM agents

We consider the counterfactual scenario where a disease peak is observed due to an initial infection seed of non-MSM agents. We run these simulations on a smaller population of 10,000 agents, of which 100 are MSMs. We find the results are qualitatively identical to the larger populations.

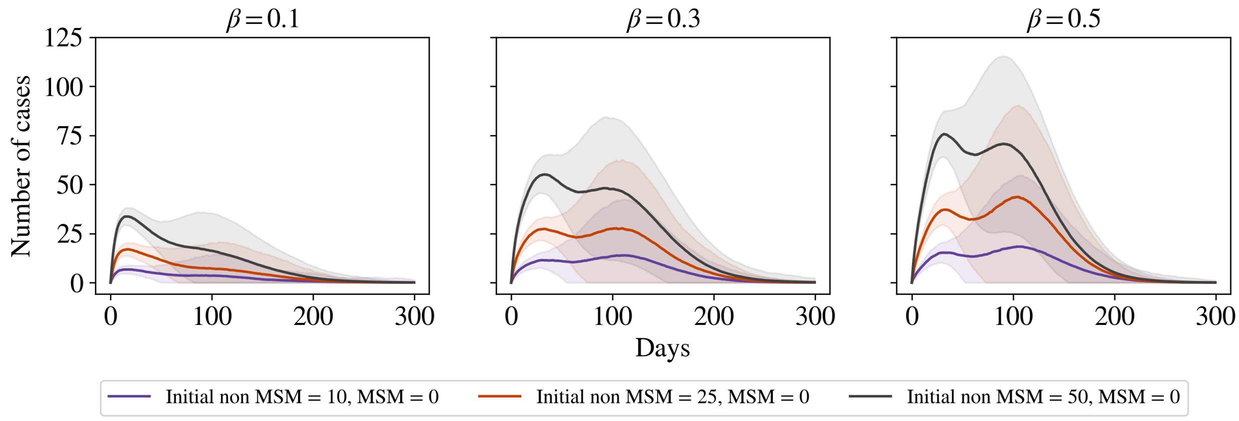

Fig S3.1: Achieving similar disease peaks with an initial non-MSM seed. We initialise infection with 10, 25, and 50 infected non-MSM agents. The broad, filled regions represent 1σ confidence intervals, indicating that entry into the MSM population is not guaranteed, especially at lower infection seeds. We stress that these results are dependent on the structure of the population. All results are averages over more than 500 stochastic runs.

We find that in order to achieve a peak in active infections that is comparable with the peak that would have occurred when the initial infection seed was 1% of MSMs, we would need to start with close to 50 non-MSM agents. These initial conditions lead to an interesting two-peak structure in the active infections. The first peak is due to infection in the non-MSM community. Given sufficient transmission, this can then cause the disease to enter the the MSM network, where it spreads much more rapidly, leading to the second, MSM-driven, peak. However, these results depend strongly on the structure of the population.
