# Supplementary material for: Simulating a potential mpox outbreak: Implications for control in non-endemic settings
Source: PLOS Glob Public Health. 2026 Jun 29;6(6):e0006630. doi: 10.1371/journal.pgph.0006630 (PMC13313346; doi:10.1371/journal.pgph.0006630)
Supplement: S4 Appendix — We study the effect of varying the frequency at which MSMs engage in sexual activity within their sexual network. We run simulations for a frequency of sexual contacts that is (i) double and (ii) half what is described in the main paper. We find, as expected, that increasing the frequency of sexual encounters effectively increases the rate of producing secondary infections. We also find that in the range considered here, the increase in frequency can be offset by a corresponding decrease in the probability of sexual transmission (i.e., μ) for the same reason. (PDF) [file pgph.0006630.s004.pdf]

S4 Appendix: Effect of sexual contact frequency on disease spread

In this section we consider the effect of varying the frequency at which MSMs engage in sexual activity within their sexual network. In the main paper we have considered that MSMs engage in a sexual encounter on average once a week, i.e. $\tau = 7$ days. (Since encounters are initiated independently per agent, this translates to each pair of MSMs who are sexual contacts of each other meeting twice a week on average.)

Here, we consider scenarios in which MSM agents engage in sexual activity (i) twice every week ($\tau = 3.5$ days, shown in Fig S4.1), and (ii) once every 2 weeks ($\tau = 14$ days, shown in Fig S4.2).

We find, as expected, that increasing the frequency of sexual encounters (i.e. reducing τ) effectively increases the reproductive ratio for sexual contacts, as the rate of creating secondary infections via sexual transmission must be proportional to the frequency with which partners meet each other. We also find that in the range considered here, the increase in frequency can be offset by a corresponding *decrease* in the probability of sexual transmission (i.e., μ) for the same reason.

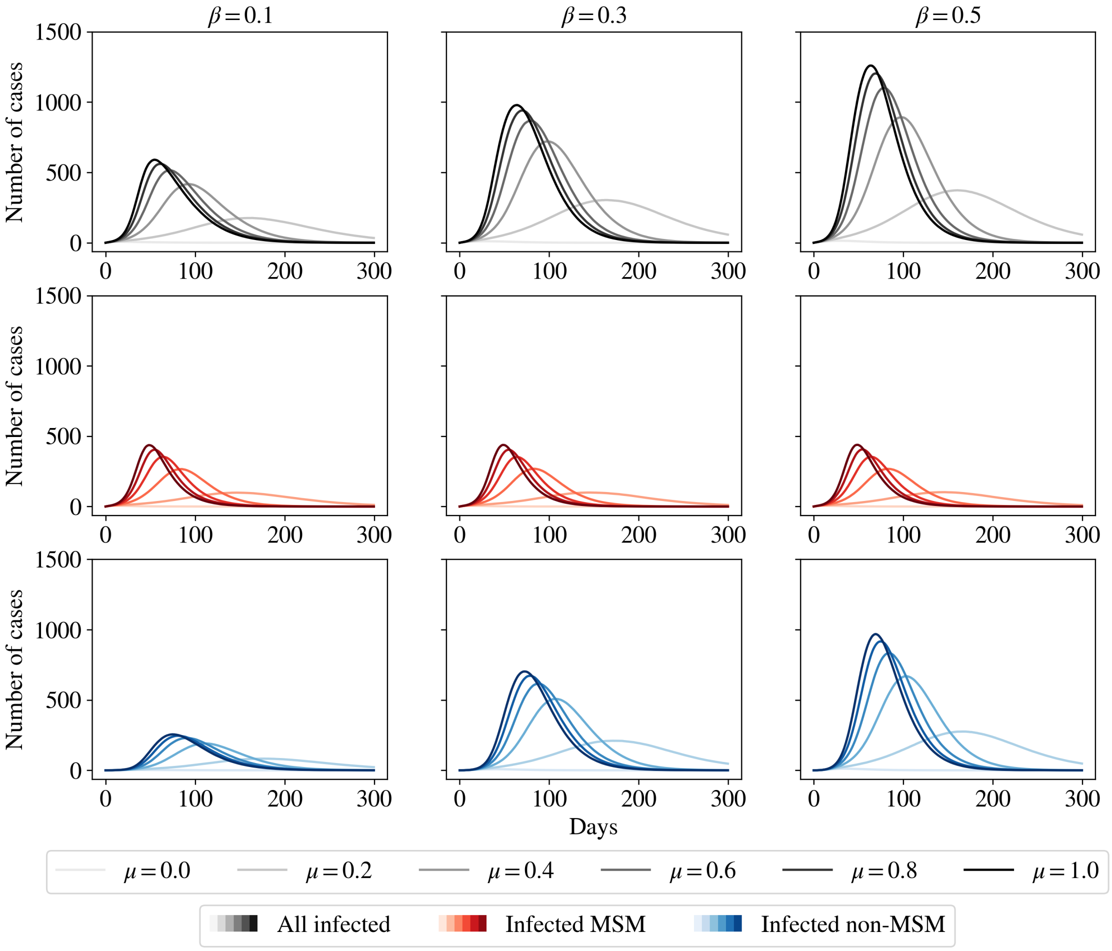

Fig S4.1: Effects of increasing frequency of sexual contacts. We re-run the same baseline runs from the main paper but increase the encounter frequency to twice a week. This leads to a much steeper rise in cases in the sexual network, which translates to an overall rise that is also steeper.

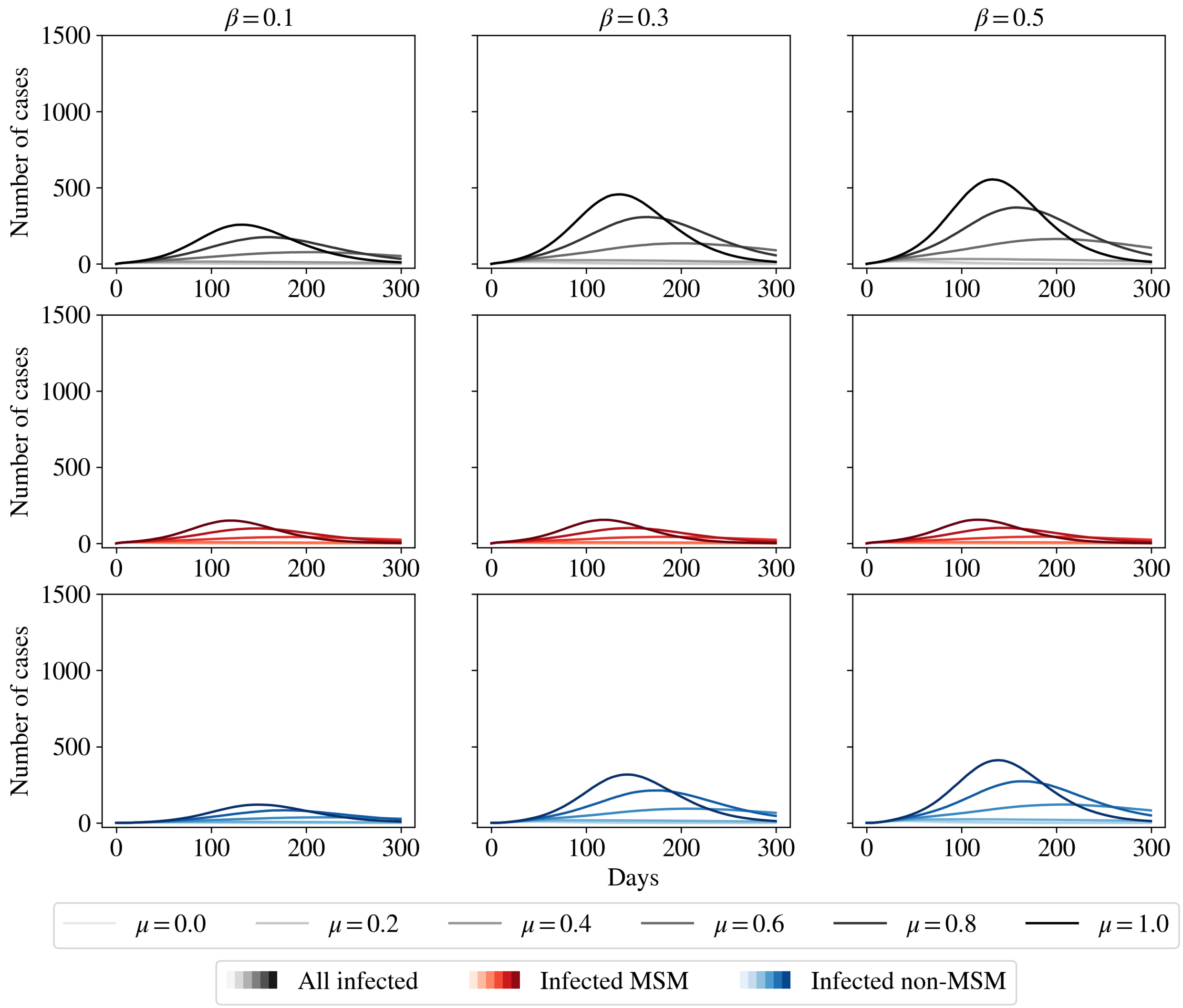

Fig S4.2: Effects of decreasing the frequency of sexual contacts. We re-run the same baseline runs from the main paper but *decrease* the meeting frequency to once in two weeks. This reduces the rise in cases in the sexual network, which consequently slows down the spread in the rest of the population.
