# Supplementary material for: Simulating a potential mpox outbreak: Implications for control in non-endemic settings
Source: PLOS Glob Public Health. 2026 Jun 29;6(6):e0006630. doi: 10.1371/journal.pgph.0006630 (PMC13313346; doi:10.1371/journal.pgph.0006630)
Supplement: S5 Appendix — We extend the vaccination analysis by considering higher daily vaccination rates with and without weak workplace transmission. We find that at sufficiently high vaccination rates the differences between strategies are effectively washed out, while lower rates substantially reduce, but do not eliminate, the infection peak. (PDF) [file pgph.0006630.s005.pdf]

S5 Appendix: Additional results for vaccination strategies

In the main paper, we considered the case when 10 vaccines (VR=1%) were available per day, and were administered to MSMs in the population. Here, we consider the situation where there are 100 vaccines per day. At this rate, all MSM agents in our population would be vaccinated in a 10-day period. The vaccine is assumed to still be a single-dose vaccine, with an efficacy of 75% in reducing probability of contracting the disease.

We recreate Fig 5 of the main paper using 100 vaccines per day (corresponding to a vaccination rate of 10% of the MSM population), as shown in Fig S5.1. We see that all effects of different vaccination strategies are washed out at these high vaccination rates.

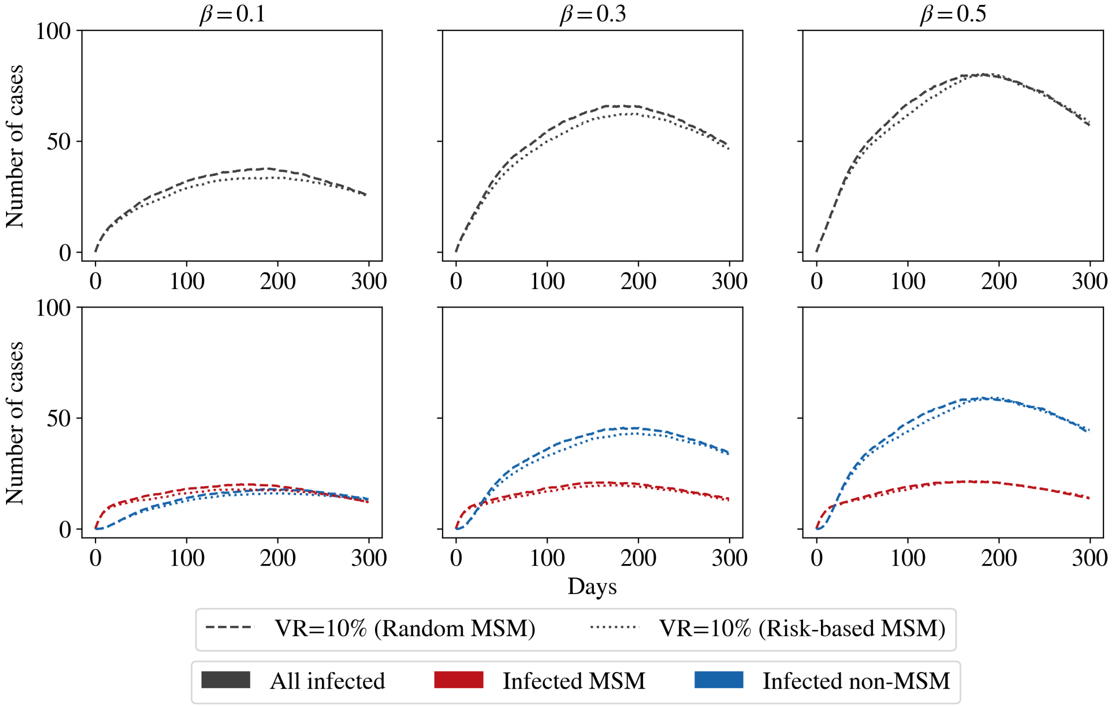

Fig S5.1: Effects of vaccination strategies on disease peak. Vaccinating 100 individuals per day is very efficient in reducing the peak of infection, with all differences in strategy being completely washed out. Again, the curves are averages over 500 runs.

We can now look at the combined effect of allowing for weak workplace transmission and a concurrent vaccination drive. In Fig S5.2 we show results for different workplace transmission factors of 0%, 1%, and 5%, alongside a concurrent vaccination drive of 10 vaccines per day (VR=1% of MSMs). We see that vaccination greatly reduces the number of cases, reducing the peak from close to 1500 cases (see Fig 7 of the main paper) to 750 cases in the worst case of a 5% workplace transmission. At higher vaccination rates of 100 vaccines per day (not shown here), the peak in active infections is effectively removed.

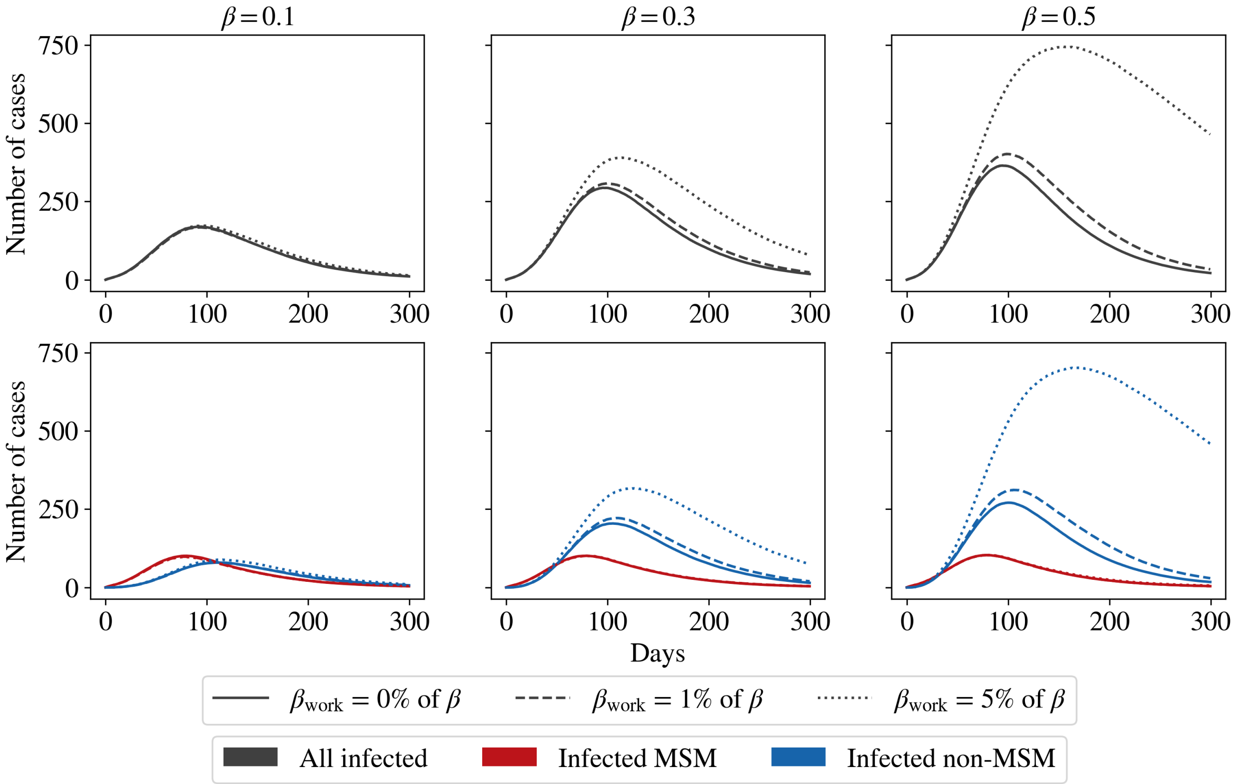

Fig S5.2: Effects of a VR=1% and workplace transmission on disease peak. A daily vaccination of 1% of the MSM population is effective at reducing the peak. However, at higher workplace transmission, a longer tail of infection persists. The curves are averages over 500 runs.
