# Supplementary material for: Simulating a potential mpox outbreak: Implications for control in non-endemic settings
Source: PLOS Glob Public Health. 2026 Jun 29;6(6):e0006630. doi: 10.1371/journal.pgph.0006630 (PMC13313346; doi:10.1371/journal.pgph.0006630)
Supplement: S6 Appendix — We examine the impact of varying self-reporting delays and contact tracing efficiencies on the effectiveness of ring vaccination. We find that higher tracing efficiency significantly reduces and delays the infection peak, while shorter self-reporting delays improve outbreak control, although with diminishing returns at longer delays. (PDF) [file pgph.0006630.s006.pdf]

S6 Appendix: Effect of varying τ_{SR} and f_{trace} in ring vaccination

To study the effect of the self-reporting rates and contact tracing efficiencies on the overall reduction in cases because of a ring vaccination strategy, we run simulations for multiple parameters: first, we set $\tau_{\text{SR}} = 1$ day (corresponding to "immediate" reporting) and run simulations for all values of f_{trace}. Next, we set $f_{\text{trace}} = 80\%$ and show the results for all mean self-reporting delays. These plots can be seen in Figs S6.1 and S6.2 respectively.

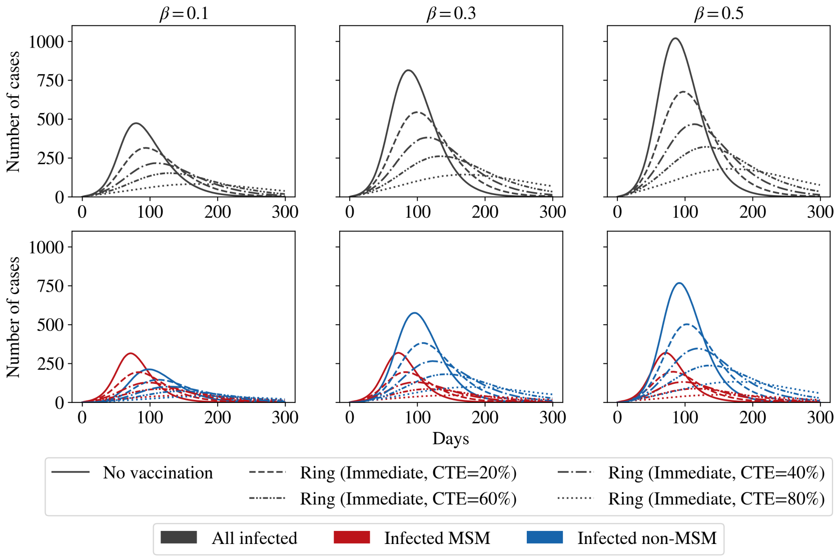

Fig S6.1: Effect of contact tracing efficiency on ring vaccination. Higher tracing efficiencies pull the peak down and shift it to later times. As before, the curves are averages over 500 runs.

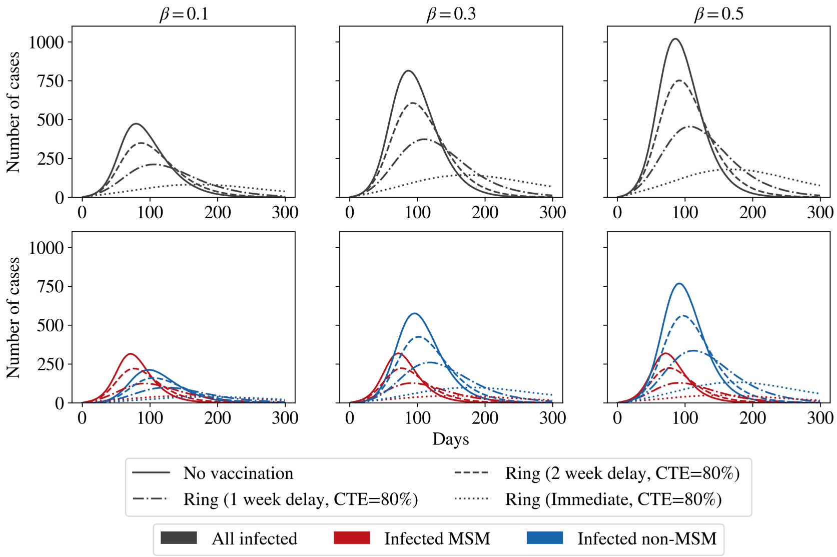

Fig S6.2: Effect of self-reporting delay on ring vaccination. The difference between immediate reporting and a 1-week delay is significant, but the effect is diminished when the delay is increased to 2 weeks. However, even at 2-week delays, 80% contact tracing efficiency can produce significant reductions in the active infections. As before, the curves are averages over 500 runs.
