# Supplementary material for: Simulating a potential mpox outbreak: Implications for control in non-endemic settings
Source: PLOS Glob Public Health. 2026 Jun 29;6(6):e0006630. doi: 10.1371/journal.pgph.0006630 (PMC13313346; doi:10.1371/journal.pgph.0006630)
Supplement: S7 Appendix — We show results for the epidemic curves with workplace transmission without sexual transmission (μ=0). We find that in all cases the disease dies out without spreading, demonstrating that the MSM subnetwork is essential for sustaining transmission in the population. Additionally, we classify active infections by infection source for the case where μ=1.0. We find that the long tail in infections is driven by workplace contacts of MSMs and their households, which allows the disease to persist even after it has died out in the high-risk MSM subnetwork. (PDF) [file pgph.0006630.s007.pdf]

S7 Appendix: Primary and secondary transmission pathways

We first study the role that transmission in the MSM sexual network has on the spread of the disease. We do this by examining the same results as in Fig 7 of the main paper, but with $\mu = 0$, effectively removing sexual contact as a possible channel for disease transmission. We see in Fig S7.1 that in all cases the disease dies out without spreading. Thus, the much smaller MSM subnetwork is essential as it, along with the long generation time, contributes to sustaining the disease in the population.

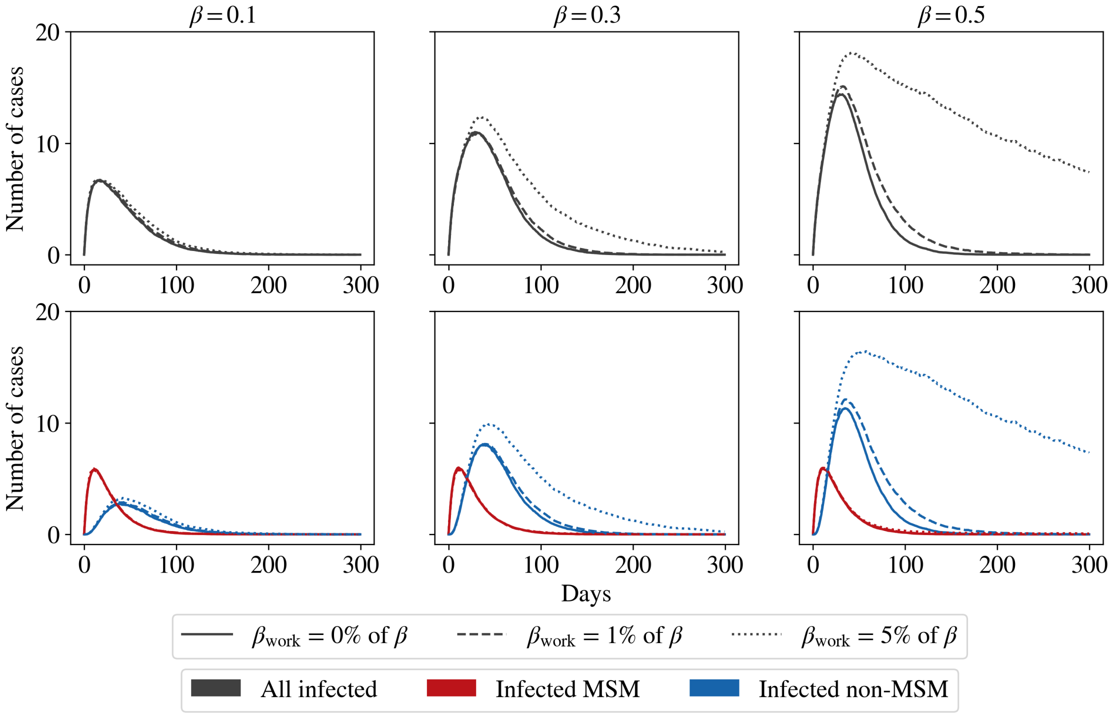

Fig S7.1: Effects of weak workplace transmission on active infections without an underlying MSM subnetwork. We repeat the simulations of Fig 7 in the main paper, but with $\mu = 0$, meaning that the disease does not spread in the MSM subnetwork. We find that in every case, the disease dies out without spreading to the general population. As before, the curves are averages over 500 runs.

Additionally, to identify the source of the long tail in infections, we further show the results of Fig 7 of the main paper, but divide the active infections into three classes: infections caused by MSMs (either through sexual transmission with their sexual contacts, or through non-sexual household transmission), infections caused by household members of MSMs, and infections caused by secondary contacts whom we identify as workplace contacts of MSMs and their household members. In Fig S7.2 we compare the cases associated with these three classes of "infectors" on the population. We see from this graph that the long tail arises almost exclusively from the secondary contacts of MSMs and their households. Allowing for a small possibility of workplace transmission thus allows the disease to escape the initially high-risk transmission network and persist through broader workplace-mediated transmission chains in the general population, which sustain long epidemic tails even after direct transmission within the MSM sub-network has declined.

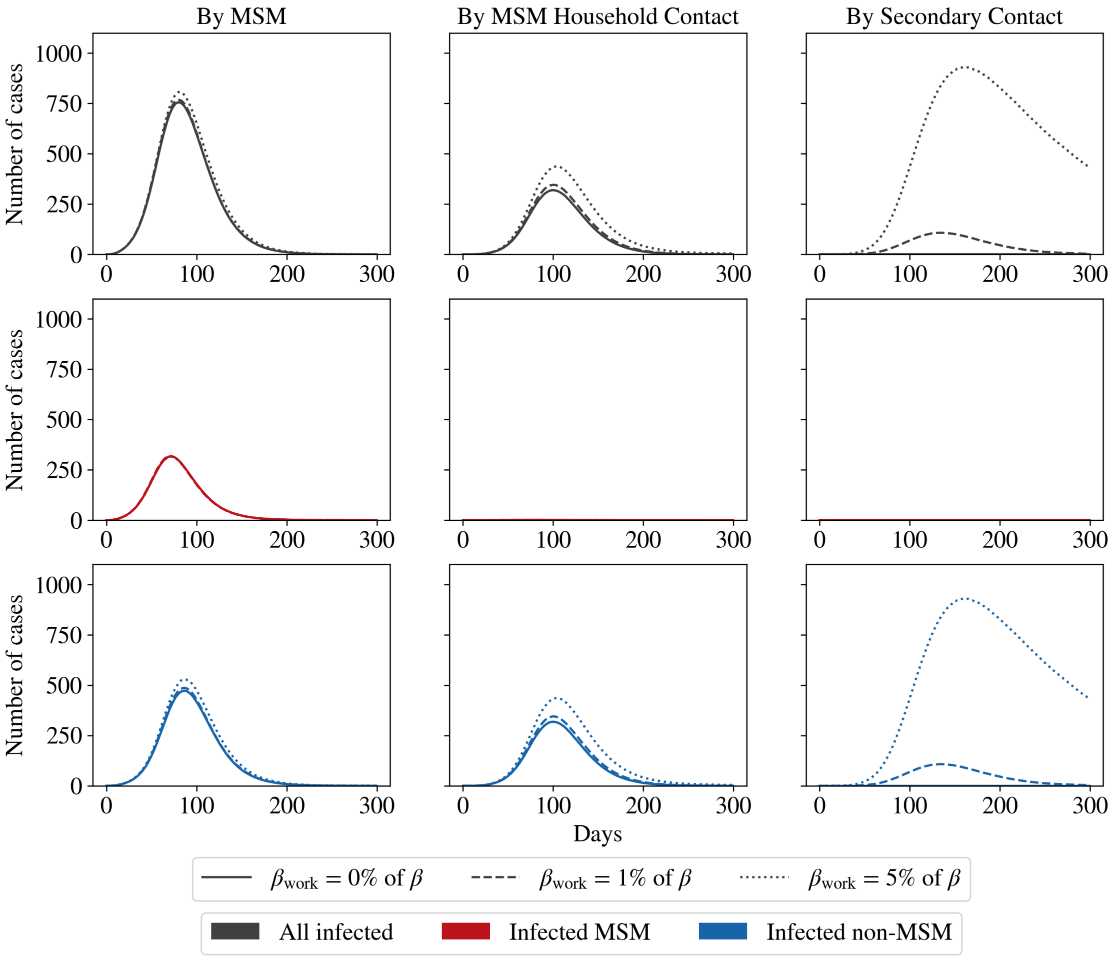

Fig S7.2: Cases categorised by infector type. We repeat the simulations of Fig 7 in the main paper, but categorise the active infections based on the type of infecting agent. We divide the population into three categories: MSMs, their household contacts, and all workplace contacts of MSMs and their households. These are represented by the three columns. For each category, we identify the cases in the total population (top row), MSM population (middle row), and non-MSM population (bottom row). We find that the long tail in infections is driven by workplace contacts of MSMs and their households, which allows the disease to persist even after it has died out in the high-risk MSM sub-network. As before, the curves are averages over 500 runs.
