# Supplementary material for: Simulating a potential mpox outbreak: Implications for control in non-endemic settings
Source: PLOS Glob Public Health. 2026 Jun 29;6(6):e0006630. doi: 10.1371/journal.pgph.0006630 (PMC13313346; doi:10.1371/journal.pgph.0006630)
Supplement: Attachment — Submitted filename: mpox-responses-to-reviewers.pdf [file pgph.0006630.s009.pdf]

Responses to Reviewers

for

"Simulating a potential mpox outbreak: Implications for outbreak control in non-endemic settings"

Philip Cherian, Gautam I. Menon

May 16, 2026

Reviewer #1:

Authors did a great as they simulated mpox scenario introduced in non-endemic country like via MSM and non-MSM networks. They have also shown the role played by the vaccination in reducing the peak of mpox.

(a) Authors did not mention the limitations/flexibility of the modeling, especially when different cut-offs are used or move. For example, if the incubation period is shorter or longer, or if the contagiousness period exceeds the timeframe set in the modeling. If events which are susceptible to strongly impact the R0 or RE and they can therefore modify the pathogen's R0 (meetings, travels, crowds, hospitalization in health facility with poor level of hygiene, etc). How would the modeling works?

We thank the referee for bringing up this important point. Our supplementary information involves some sensitivity analyses. In particular, in Appendix S1, we look at the effect of varying the cutoff on our power-law-distributed sexual-contact distribution. In response to a different referee question, we have now also included an analysis on how the frequency of sexual contact affects the disease trajectory. We can easily vary both incubation and infectious periods in our simulations, but have chosen to work with point estimates for these to avoid proliferation of relatively less-known parameters.

Regarding the referee's point on external events that might strongly affect the transmission of the disease, we point out that agent-based models are particularly suited to analyse such situations. Indeed, in the original BharatSim paper, as well as the BharatSim documentation, we show how modellers can easily incorporate multiple interventions quite simply. We can model lockdowns, vaccine programs, multiple strains, school closures and a host of other measures, as shown there, but also the $\mathcal{R}_0$ modifying events that the referee lists. In this paper, we choose not to do them, since data on these are limited and country-specific. These could easily be adapted to address changes in effective $\mathcal{R}_0$. We have provided a detailed discussion of other limitations to our model as well. Finally, a related change is that we have added some text to the discussion session to reflect new directions that the modelling could take, in India, where the interactions between MSM and transgender/transsexual (Hijra) communities adds a layer of complex social behaviour.

We have added the following paragraph to the paper to address the limitations and flexibility of our model:

Our networks are static and not dynamic, but some degree of dynamicity is to be expected, as members of the MSM population migrate in and out of the overall population, as well

as establish new connections within pre-existing networks. Agent-based models are powerful because they can be embedded into specific geographies, allowing for a description of physical hotspots of interaction, including casual sexual encounters. However, this would involve, at least for now, far more assumptions regarding the dynamical character of such MSM sexual networks – we defer these questions to future work. In this study, we modelled the latent period, infectious period, and self-reporting delay using exponential distributions, an assumption that simplifies the dynamics but does not fully capture the heterogeneity observed in clinical data. A natural extension of this work would be to incorporate empirically informed distributions for disease progression and reporting delays, which are often better described by lognormal distributions.

(b) Authors should clearly state out (summarize) the public health implications of this tool in real settings. They should also elaborate more on how this should practically be used and/or adapted to other health emergencies (outbreaks).

We agree, and we have modified the main paper to reflect this. We show that allowing for spread outside the MSM subnetwork can lead to a long tail in time, which in turn can lead to many more infections in the non-MSM network. By decomposing the sources of infection across the coupled networks, we are also able to evaluate where differential interventions can make a difference. We show how a better knowledge of the MSM network as well as the contacts that MSMs have with non-MSMs can be used to target interventions and assess their impact.

The following paragraph in the revised manuscript addresses this:

The work described here models how infections can spread in non-endemic settings, showing how, depending on the efficiency of transmission in the MSM subnetwork and the broader contact network, policies for outbreak control may have to be applied to the larger contact network of MSMs. We found that restricting transmission to the household expands the numbers of infected. Adding the possibility of workplace transmission increases this number further, provided the rate of workplace transmission of infection is sufficiently high. Interestingly, these two modes of transmission, MSM and non-MSM, interact reciprocally to produce complex temporal behaviour [57]. While the disease cannot spread in purely non-MSM networks, since the relevant $\mathcal{R}_0$ is sub-threshold, the epidemic can be sustained through the interaction between networks.

(c) In countries with no restrictions of homosexuals' rights (MSM can easily show up), how would the modeling fit for mpox cases? Because the size of MSM networks and contacts can substantially increase, despite the poor mpox prevalence noted.

Our study is specifically appropriate to the Indian population, because of our choice of the synthetic population that enters our model. The prevalence of mpox cases depends strongly not only on the number of MSM agents, but also on the underlying sexual network, contact frequency, and mixing patterns. The extent to which these networks are a matter of public health knowledge, of course, differs from country to country, depending on the costs assigned to the visibility of homosexual behaviour. We believe that our study underscores the importance of collecting further data on such networks in India. In principle, our model can also describe networks in which only a fraction of nodes are observable, accounting for apparent restricted spread among the population. Better input from surveys which quantify reporting behaviour would help us parametrize this better.

Our model can be easily generalised to different countries in which the disease is non-endemic, provided data is available to construct a realistic population.

(d) It is known that some MSM can also be heterosexual (bisexual). In this condition, I will suggest authors adding this case-study into the modeling and show how this affects predictions?

We thank the referee for suggesting this important point. In our study, we have assumed that MSMs can transmit the disease through one of two channels: transmission through sexual contact, or weak transmission between family members. We have now improved and extended our model to include the possibility of bisexual MSMs who can transmit the disease to female sexual partners, thus allowing for potential heterosexual routes of transmission.

We have added the following paragraph to the main paper:

Nevertheless, studies have shown that close to a third of the MSM population is bisexual [50,51]. Of these, nearly 75% have a predominant female partner who is their wife. Consequently, we have also run simulations in which 33% of MSMs are bisexual. For these bisexual MSM agents, in addition to their MSM contacts drawn from the distribution as described earlier, a single female household member is further designated as their female sexual partner. The results of these simulations are shown in Appendix S2.

Briefly, our results show that the introduction of bisexual MSMs with female sexual partners from their household can slow the spread within the MSM sexual network by reducing the possibility of MSM-MSM contact. We find that this corresponds to a slower spread in the non-MSM network as well, however for low values of household transmission (β) and high values of transmission per sexual encounter (μ) this can actually increase the number of non-MSM agents infected as it effectively increases the household attack rate in MSM households.

(e) I think sexual behaviors should be presented separately depending on whether this could be anal (high risk for mpox transmission) and vaginal. We assume from the literature that the risk should not be the same in both circumstances. This can be reflected into the modeling to capture essential nuances, especially for public health decision-making.

In our study, we have assumed that sexual transmission occurs only between MSMs. While this is an assumption, we believe that it is valid as a US CDC report showed that among mpox cases in the United States, 99% occurred in men, with 94% reporting recent MSM activity (see Philpott D, Hughes CM, Alroy KA, et al. Epidemiologic and Clinical Characteristics of Monkeypox Cases — United States, May 17–July 22, 2022. MMWR Morb Mortal Wkly Rep 2022;71:1018-1022. DOI: `http://dx.doi.org/10.15585/mmwr.mm7132e3`).

As described in the previous response, in Appendix S2 of our revised manuscript we address the possibility of vaginal transmission at an enhanced rate, by allowing members of the MSM population to contact one (female) household member.

Reviewer #2:

(a) The author did a relevant review of literature which is demonstrated by referencing necessary past research.

We thank the reviewer for their positive assessment of our literature review.

(b) The author has clearly stated that all data is available freely on GitHub though link to the Github repository was not provided.

This was an oversight on our part; we have now explicitly included the link to the GitHub repository in the main paper. The following line has been added to the revised manuscript: *The model and associated population files have been made available on the GitHub repository* *`https://github.com/dpcherian/mpox-model`.*

(c) Author used BharatSim to develop the agent-based model. BharatSim has been clearly demonstrated in other research papers to be able to construct agent-based models for infectious disease, validating the framework.

(d) Assumptions in constructing model are well explained. However, it is not clear how the author deduced:

- Transmission rates between MSM
- Transmission rates between non MSM
- Transmission rates between MSM and non MSM
- The number of sexual partners.

We vary the per-encounter infection probability for MSM-sexual interactions (the parameter μ in the main paper) to account for different transmission rates between MSMs. These will be functions of contact rates as well as transmissibility, e.g. direct MSM sexual contact, MSM-non-MSM heterosexual contacts, MSM-non-MSM contact via fomites and finally non-MSM to non-MSM transmission. While MSM-MSM and MSM-heterosexual partner infection probabilities are known to some degree, we know very little about the others, so we have chosen to model an appropriate and reasonable range. For the moment, it is hard to see how we could do this better in the absence of data that can better quantify these.

(e) While the non-endemic country is well stated (India) but the model seems not to consider geographical disparity of MSM. The author might want to consider that MSM are usually clustered in particular areas with certain characteristics. this uneven distribution of MSM might affect the outbreak pattern.

In our model, the quantity of interest is the network of sexual interactions. This network does indeed sits on an underlying geography, since agents are assigned home and work locations. We agree that popular meeting places for sexual encounters, for example, can only be incorporated in explicit spatial descriptions. These are valuable to understand in the context of potential interventions. However, a network description of the interactions which lead to infections and potential outbreaks is the appropriate underlying description, as has been argued extensively.

(f) The rationale behind using the truncated power law distribution has not been clearly defined, please could you clarify why you preferred to use the truncated power law distribution.

In this work, we used a truncated power law because studies have shown that sexual contact networks exhibit strong heterogeneity with a small number of highly connected individuals, but finite population and behavioural constraints impose limits on the range of number of sexual partnerships. We have now addressed this explicitly in the paper, saying:

We choose a truncated power-law distribution because empirical studies of MSM sexual-contact networks consistently report substantial heterogeneity in the number of sexual partners, with most individuals having relatively few partners and a small fraction having disproportionately many [7,42]. A power-law form captures this heavy-tailed structure, which is important for representing heterogeneous transmission and potential super-spreading dynamics. Additionally, we impose lower and upper cut-offs to reflect the finite range of partner numbers observed empirically in Ref. [33]. The lower and upper cut-offs therefore represent the observed range of the available data.

(g) Since the frequency of sexual contact is an important parameter limiting it to once a week could be crucial, has the author considered varying this value within a logical range.

We thank the referee for bringing this up, and we agree that including this in our sensitivity analyses is important. We have run our baseline scenarios for two cases, when the average frequency of sexual contact is twice a week, and once in two weeks. We find no qualitative changes in our results: a higher meeting rate causes the disease to spread more rapidly amongst the MSM network, and can be compared to an effective increase in the sexual transmission probability μ. This work has now been included in the Supplementary Information.

(h) Conclusions are stated based on findings from the work

(i) The Author has clearly stated limitations of the work.

(j) English language was used in writing the manuscript, with well constructed sentences and proper use of punctuations.

Finally, to address comments from both reviewers, in the revised manuscript, we address some further limitations of our work. Given that sexual contact networks are inadequately known, the applicability of our results is limited to those cases where the networks we consider are adequate representations. Because these networks derive from real data from MSM communities in an Indian state, they may not have broad generality. We do not implement the complex nature of sexual communities and interactions, such as the role of transgender communities in the Indian context. Rather than well-defined contacts which enter as nodes and links between them, the real situation may also involve transient and anonymous MSM interactions, adding more complex dynamics to the static situation we model. Clearly this is an important area for further work.
