# Supplementary material for: Simulating a potential mpox outbreak: Implications for control in non-endemic settings
Source: PLOS Glob Public Health. 2026 Jun 29;6(6):e0006630. doi: 10.1371/journal.pgph.0006630 (PMC13313346; doi:10.1371/journal.pgph.0006630)
Supplement: Peer review history

# Peer review: Decision Letter 0

**DOI:** 10.1371/journal.pgph.0006630.r001

**Authors:** Andrés Henao-Martínez

6 Apr 2026

PGPH-D-26-00719

Simulating a potential mpox outbreak in India: Implications for outbreak control in non-endemic settings

PLOS Global Public Health

Dear Dr. Menon,

Thank you for submitting your manuscript to PLOS Global Public Health. After careful consideration, we feel that it has merit but does not fully meet PLOS Global Public Health’s publication criteria as it currently stands. Therefore, we invite you to submit a revised version of the manuscript that addresses the points raised during the review process.

Please submit your revised manuscript by May 21 2026 11:59PM. If you will need more time than this to complete your revisions, please reply to this message or contact the journal office at globalpubhealth@plos.org. When you're ready to submit your revision, log on to https://www.editorialmanager.com/pgph/ and select the 'Submissions Needing Revision' folder to locate your manuscript file.

Please include the following items when submitting your revised manuscript:

- A letter that responds to each point raised by the editor and reviewer(s). You should upload this letter as a separate file labeled 'Response to Reviewers'.
- A marked-up copy of your manuscript that highlights changes made to the original version. You should upload this as a separate file labeled 'Revised Manuscript with Track Changes'.
- An unmarked version of your revised paper without tracked changes. You should upload this as a separate file labeled 'Manuscript'.

Guidelines for resubmitting your figure files are available below the reviewer comments at the end of this letter.

As the corresponding author, your ORCID iD is verified in the submission system and will appear in the published article. PLOS supports the use of ORCID, and we encourage all coauthors to register for an ORCID iD and use it as well. Please encourage your coauthors to verify their ORCID iD within the submission system before final acceptance, as unverified ORCID iDs will not appear in the published article. *Only*  the individual author can complete the verification step; PLOS staff *cannot*  verify ORCID iDs on behalf of authors.

We look forward to receiving your revised manuscript.

Kind regards,

Andrés F. Henao-Martínez, M.D.

Academic Editor

PLOS Global Public Health

Journal Requirements:

1. Please note that PLOS Global Public Health has specific guidelines on code sharing for submissions in which author-generated code underpins the findings in the manuscript. In these cases, all author-generated code must be made available without restrictions upon publication of the work. Please review our guidelines at https://journals.plos.org/globalpublichealth/s/materials-and-software-sharing#loc-sharing-code and ensure that your code is shared in a way that follows best practice and facilitates reproducibility and reuse.

2. Please amend your detailed Financial Disclosure statement. This is published with the article. It must therefore be completed in full sentences and contain the exact wording you wish to be published.

a. Please clarify all sources of funding (financial or material support) for your study. List the grants (with grant number) or organizations (with url) that supported your study, including funding received from your institution.

b. State the initials, alongside each funding source, of each author to receive each grant.

c. State what role the funders took in the study. If the funders had no role in your study, please state: “The funders had no role in study design, data collection and analysis, decision to publish, or preparation of the manuscript.”

d. If any authors received a salary from any of your funders, please state which authors and which funders.

3. We ask that a manuscript source file is provided at Revision. Please upload your manuscript file as a .doc, .docx, .rtf or .tex.

4. Please provide separate figure files in .tif or .eps format.

For more information about figure files please see our guidelines:  LINK

https://journals.plos.org/globalpublichealth/s/figures

https://journals.plos.org/globalpublichealth/s/figures#loc-file-requirements

If the reviewer comments include a recommendation to cite specific previously published works, please review and evaluate these publications to determine whether they are relevant and should be cited. There is no requirement to cite these works unless the editor has indicated otherwise.

Additional Editor Comments (if provided):

[Note: HTML markup is below. Please do not edit.]

Reviewers' comments:

Reviewer's Responses to Questions

**Comments to the Author**

1. Does this manuscript meet PLOS Global Public Health’s publication criteria? Is the manuscript technically sound, and do the data support the conclusions? The manuscript must describe methodologically and ethically rigorous research with conclusions that are appropriately drawn based on the data presented.

Reviewer #1: Yes

Reviewer #2: Yes

**********

2. Has the statistical analysis been performed appropriately and rigorously?

Reviewer #1: Yes

Reviewer #2: I don't know

**********

3. Have the authors made all data underlying the findings in their manuscript fully available (please refer to the Data Availability Statement at the start of the manuscript PDF file)?

The PLOS Data policy requires authors to make all data underlying the findings described in their manuscript fully available without restriction, with rare exception. The data should be provided as part of the manuscript or its supporting information, or deposited to a public repository. For example, in addition to summary statistics, the data points behind means, medians and variance measures should be available. If there are restrictions on publicly sharing data—e.g. participant privacy or use of data from a third party—those must be specified.

Reviewer #1: Yes

Reviewer #2: Yes

**********

4. Is the manuscript presented in an intelligible fashion and written in standard English?

PLOS Global Public Health does not copyedit accepted manuscripts, so the language in submitted articles must be clear, correct, and unambiguous. Any typographical or grammatical errors should be corrected at revision, so please note any specific errors here.

Reviewer #1: Yes

Reviewer #2: Yes

**********

5. Review Comments to the Author

Please use the space provided to explain your answers to the questions above. You may also include additional comments for the author, including concerns about dual publication, research ethics, or publication ethics. (Please upload your review as an attachment if it exceeds 20,000 characters)

Reviewer #1: Simulating a potential mpox outbreak in India: Implications for outbreak control in nonendemic settings

Authors did a great as they simulated mpox scenario introduced in non-endemic country like via MSM and non-MSM networks. They have also shown the role played by the vaccination in reducing the peak of mpox.

Number the lines of the manuscript throughout the document

1) Authors did not mention the limitations/flexibility of the modeling, especially when different cut-offs are used or move. For example, if the incubation period is shorter or longer, or if the contagiousness period exceeds the timeframe set in the modeling. If events which are susceptible to strongly impact the R0 or RE and they can therefore modify the pathogen’s R0 (meetings, travels, crowds, hospitalization in health facility with poor level of hygiene, etc). How would the modeling works?

2) Authors should clearly state out (summarize) the public health implications of this tool in real settings. They should also elaborate more on how this should practically be used and/or adapted to other health emergencies (outbreaks).

3) In countries with no restrictions of homosexuals’ rights (MSM can easily show up), how would the modeling fit for mpox cases? Because the size of MSM networks and contacts can substantially increase, despite the poor mpox prevalence noted.

4) It is known that some MSM can also be heterosexual (bisexual). In this condition, I will suggest authors adding this case-study into the modeling and show how this affects predictions?

5) I think sexual behaviors should be presented separately depending on whether this could be anal (high risk for mpox transmission) and vaginal. We assume from the literature that the risk should not be the same in both circumstances. This can be reflected into the modeling to capture essential nuances, especially for public health decision-making.

Reviewer #2: 1. The author did a relevant review of literature which is demonstrated by referencing necessary past research.

2. The author has clearly stated that all data is available freely on GitHub though link to the Github repository was not provided.

3. Author used BharatSim to develop the agent-based model. BharatSim has been clearly demonstrated in other research papers to be able to construct agent-based models for infectious disease, validating the framework.

4. Assumptions in constructing model are well explained

However, it is not clear how the author deduced;

• Transmission rates between MSM

• Transmission rates between non MSM

• Transmission rates between MSM and non MSM

• The number of sexual partners.

5. While the non-endemic country is well stated (India) but the model seems not to consider geographical disparity of MSM. The author might want to consider that MSM are usually clustered in particular areas with certain characteristics. this uneven distribution of MSM might affect the outbreak pattern.

6. The rationale behind using the truncated power law distribution has not been clearly defined, please could you clarify why you preferred to use the truncated power law distribution.

7. Since the frequency of sexual contact is an important parameter limiting it to once a week could be crucial, has the author considered varying this value within a logical range.

8. Conclusions are stated based on findings from the work

10. The Author has clearly stated limitations of the work.

11. English language was used in writing the manuscript, with well constructed sentences and proper use of punctuations.

**********

6. PLOS authors have the option to publish the peer review history of their article (what does this mean?). If published, this will include your full peer review and any attached files.

**Do you want your identity to be public for this peer review?**  If you choose “no”, your identity will remain anonymous but your review may still be made public.

For information about this choice, including consent withdrawal, please see our Privacy Policy.

Reviewer #1: No

Reviewer #2: **Yes:** Fally Terry Bealy

**********

[NOTE: If reviewer comments were submitted as an attachment file, they will be attached to this email and accessible via the submission site. Please log into your account, locate the manuscript record, and check for the action link "View Attachments". If this link does not appear, there are no attachment files.]

-->Figure Resubmissions:

While revising your submission, we strongly recommend that you use PLOS’s NAAS tool (https://ngplosjournals.pagemajik.ai/artanalysis) to test your figure files. NAAS can convert your figure files to the TIFF file type and meet basic requirements (such as print size, resolution), or provide you with a report on issues that do not meet our requirements and that NAAS cannot fix.

After uploading your figures to PLOS’s NAAS tool - https://ngplosjournals.pagemajik.ai/artanalysis, NAAS will process the files provided and display the results in the "Uploaded Files" section of the page as the processing is complete. If the uploaded figures meet our requirements (or NAAS is able to fix the files to meet our requirements), the figure will be marked as "fixed" above. If NAAS is unable to fix the files, a red "failed" label will appear above. When NAAS has confirmed that the figure files meet our requirements, please download the file via the download option, and include these NAAS processed figure files when submitting your revised manuscript.

# Author comment: Author response to Decision Letter 1

**DOI:** 10.1371/journal.pgph.0006630.r002

24 May 2026

Attachment Submitted filename: mpox-responses-to-reviewers.pdf

# Editor report: Decision Letter 1

**DOI:** 10.1371/journal.pgph.0006630.r003

**Authors:** Andrés Henao-Martínez, Andrés Henao-Martínez

26 May 2026

Simulating a potential mpox outbreak: Implications for outbreak control in non-endemic settings

PGPH-D-26-00719R1

Dear Prof. Menon,

We are pleased to inform you that your manuscript 'Simulating a potential mpox outbreak: Implications for outbreak control in non-endemic settings' has been provisionally accepted for publication in PLOS Global Public Health.

Before your manuscript can be formally accepted you will need to complete some formatting changes, which you will receive in a follow up email. A member of our team will be in touch with a set of requests.

Please note that your manuscript will not be scheduled for publication until you have made the required changes, so a swift response is appreciated.

IMPORTANT: The editorial review process is now complete. PLOS will only permit corrections to spelling, formatting or significant scientific errors from this point onwards. Requests for major changes, or any which affect the scientific understanding of your work, will cause delays to the publication date of your manuscript.

If your institution or institutions have a press office, please notify them about your upcoming paper to help maximize its impact. If they'll be preparing press materials, please inform our press team as soon as possible -- no later than 48 hours after receiving the formal acceptance. Your manuscript will remain under strict press embargo until 2 pm Eastern Time on the date of publication. For more information, please contact globalpubhealth@plos.org.

Thank you again for supporting Open Access publishing; we are looking forward to publishing your work in PLOS Global Public Health.

Best regards,

Andrés F. Henao-Martínez, M.D.

Academic Editor

PLOS Global Public Health

***********************************************************

Reviewer Comments (if any, and for reference):
